# Secondary care clinicians and staff have a key role in delivering equivalence of care for prisoners: A qualitative study of prisoners’ experiences

**DOI:** 10.1016/j.eclinm.2020.100416

**Published:** 2020-06-21

**Authors:** Chantal Edge, Mr Rich Stockley, Mrs Laura Swabey, Mrs Emma King, Mr Fabien Decodts, Dr Jake Hard, Dr Georgia Black

**Affiliations:** a UCL Collaborative Centre for Inclusion Health, Institute of Epidemiology and Health Care, Room 350, 1-19 Torrington Place, Fitzrovia, London WC1E 7HB, UK; b Surrey County Council Public Health, County Hall, Penrhyn Road, Kingston upon Thames KT1 2DW, UK; c Surrey Heartlands Health and Care Partnership, Guildford and Waverley CCG, Dominion House, Woodbridge Road, Guildford, Surrey GU1 4PU, UK; d Insight and Feedback Team, Nursing Directorate, NHS England and NHS Improvement, Skipton House, 80 London Road, London SE1 6LH, UK; e UCL Behavioural Science and Health, Institute of Epidemiology and Health Care, 1-19 Torrington Place, Fitzrovia, London WC1E 7HB, UK; f Patient representative, 104 Cavatina Point, 3 Dancers Way, Greenwich, SE8 3FG, UK; g Royal College of General Practitioners Secure Environments Group, 30 Euston Square, London NW1 2FB, UK; h UCL Department of Applied Health Research, Institute of Epidemiology and Health Care, 1-19 Torrington Place, Fitzrovia, London WC1E 7HB, UK

## Abstract

Background: While challenging to provide, prisoners are entitled to healthcare equivalent to community patients. This typically involves them travelling to hospitals for secondary care, whilst adhering to the prison's operational security constraints. Better understanding of equivalence issues this raises may help hospitals and prisons consider how to make services more inclusive and accessible to prisoners.  We used prisoners’ accounts of secondary care experiences to understand how these relate to the principle of healthcare equivalence.

Methods: We undertook a qualitative interview (*n* = 17) and focus group (*n* = 5) study in the English prison estate. Prisoners who had visited acute hospitals for consultations were eligible for participation. They were recruited by peer researchers. 45 people (21 female, 24 male, average age 41) took part across five prisons. Participants were purposively recruited for diversity in gender, age and ethnicity.

Findings: Experiences of hospital healthcare were analysed for themes relating to the principle of ‘equivalence of care’ using Framework Analysis. Participants described five experiences challenging ‘equivalence of care’ for prisoners: (1) Security overriding healthcare need or experience (2) Security creating public humiliation and fear (3) Difficulties relating to prison officer's role in medical consultations (4) Delayed access due to prison regime and transport requirements and (5) Patient autonomy restricted in management of their own healthcare.

Interpretation: Achieving equivalence of care for prisoners is undermined by fear, stigma, reduced autonomy and security requirements.  It requires co-ordinated action from commissioners, managers, and providers of prison and healthcare systems to address these barriers. There is a need for frontline prison and healthcare staff to address stigma and ensure they understand common issues faced by prisoners seeking to access healthcare, while developing strategies which empower the autonomy of prisoners’ healthcare decisions.

## 1 Introduction

Prisoners are subject to health inequalities, experiencing poorer health, outcomes and access to care than the general population, [1]. For nearly 40 years, the importance of achieving a healthcare service for prisoners that is deemed ‘equivalent’ to that available in the community has been an international ethical and moral principle [2–4]. There is an international consensus that restriction of access to appropriate healthcare should not be part of the deprivation of liberty imposed as punishment. Indeed, in the 1976 landmark Estelle v. Gamble case [32], the US Supreme Court ruled that inadequate healthcare provision to prisoners was a violation of the Constitution's Eighth Amendment against cruel and unusual punishment. The way in which the state treats people in prison reflects the fundamental justice within our society [32].

Prisons can in fact provide an opportunity to address health inequalities seen in the patient population. In England and Wales we have already attempted to address equivalence by providing services inside prison that are not widely available in community primary care settings (e.g. substance misuse, blood-borne virus and mental health provisions), in order to address the most prevalent issues. Resulting benefits will be transmitted to the wider community on release [5] and contribute to a reduction in social inequalities in health. Investing in prison healthcare and striving for ‘equivalence’ therefore aims to both achieve equivalent health outcomes for prisoners and invest in the health of our society [6, 7].

In the UK, healthcare for prisoners has been provided by the National Health Services (NHS) since 2006 [8]. The presence of this free-at-the-point of access healthcare service in the UK ensures there exists largely equal access to healthcare independent of a person's status. The NHS Constitution provides the guiding principles behind the creation of the NHS, with these values also pertinent to the secure and detained population in England and Wales.

People in prison continue to access the majority of their secondary care through external hospital services. This results in additional challenges for the healthcare providers as they must work together with prison authorities in order to provide access to these external appointments. Policy makers have conflicting views about how to overcome these challenges according to principles of equivalence in order to achieve improved health outcomes. There is disagreement about whether equivalence relates to access, outcomes, or basic needs, and the closed, complex, resource-poor, prison environment is positioned as an insurmountable challenge [9]. A review of UK prison healthcare studies found there was a lack of evidence on how to improve health outcomes for prisoners by focussing on equivalence of care, and highlighted the importance of acknowledging the views of prisoners [5].

## 2 Methods

### 2.1 Study design

We performed qualitative analysis of interviews and focus groups with prisoners who have experience of accessing hospital healthcare from prison. We collaborated with peer researchers from an established prison charity during preparation, design and data collection.

### 2.2 Recruitment

Peer researchers selected participants through purposive and snowball sampling. Participants known to have experience of secondary healthcare were approached in person with a leaflet explaining study purpose and activities, supported by a verbal explanation from peers. Peers in prison known to the peer researchers were also asked to identify people who may be appropriate and willing to participate in focus groups. Focus group participants were also invited to attend 1:1 interviews. Of 29 total focus group participants, 5 took part in 1:1 interviews, with additional interview participants recruited through peer researchers’ networks at prison sites.

### 2.3 Participants

Focus groups (*n* = 5) and 1:1 interviews (*n* = 17) were undertaken by peer researchers to collect qualitative data pertaining to experiences of accessing secondary care whilst incarcerated. (10) Focus group size ranged from three to nine people, and lasted between one and two hours. All focus groups and interviews took place within prison in a neutral space.

Forty-five participants took part in the study (Table 1). Several participants (*n* = 7) dropped out or could not participate. Reasons included: other commitments (e.g. hospital attendance, gym session), receipt of bad news prior to interview, lack of prison escort to bring participant to interview, and rumours that research results would be sent to government immigration agencies.

**Table. 1:** Participant characteristics.

Total participants		45
	Participants (interviews)	17
	Participants (focus groups)	29
Gender	Male	21
	Female	24
Ethnicity	Asian	6
	Black	12
	Dual heritage*	6
	White	12
	Other	3
	Unknown	5
Age	Age range	23–69
	Mean	41

⁎ Dual heritage refers to participants who have parents from different ethnic or cultural backgrounds.

### 2.4 Data collection

Five different English prisons comprising male, female and foreign national prisoners were involved in data collection. Prisons were selected to ensure a mixture of participants both by sex, but also by sentence type (remand and sentenced prisoners). Prisons with which the prison charity had existing relationships were targeted to ensure access would likely be granted for research. Focus groups were used to understand broad issues relating to accessing secondary care in prisons, which preceded a further stage of 17 1:1 qualitative interviews with prisoners.

Semi–structured interview guides were developed with peer researchers from an established prison charity for both stages, which were reviewed by a community forum comprised of ex-offenders prior to use. Topic guides were split into three overall sections: Attending hospital from prison; Patient journey in the prison context; Leaving prison. Examples of high-level topics covered are shown in Supplementary Table1.

Throughout the data collection process, peer researchers, supported by lead researchers, reflected on what worked to gain the most accurate data from participants and adapted their questioning style appropriately.

### 2.5 Ethics

This study received ethical approval from the Camberwell St Giles NHS Research Ethics Committee(18/LO/0643) and the HMPPS National Research Committee(NRC 2018-212).

### 2.6 Informed consent

Written informed consent was obtained from all participants by peer researchers.

### 2.7 Analysis and reporting

Data collection activities were recorded on encrypted digital voice recorders and professionally transcribed. Participants did not review or comment on transcripts due to the practical constraints of reconvening participants. Instead, after independent coding of transcripts, researchers, peer researchers and prison healthcare staff met to discuss codes and verify they were reflective of the data attributed to them. During focus groups, researchers made field notes to provide context to the analysis. Focus group and interview data were subject to Framework Analysis [11] Framework Analysis involves a five step process: 1. familiarisation; 2. identifying a thematic framework; 3. indexing; 4. charting; and 5. mapping and interpretation [11]

An initial rapid review of transcripts was undertaken by five researchers (CE,GB,RS,LS,EK) to derive codes from the data to develop a coding schema, after which further in-depth re-coding was undertaken. The resulting qualitative data collected was coded using open and axial coding in Excel. For this project, axial codes related to chronological stages of secondary care appointments, and open codes were grouped according to their stage in the healthcare journey, for example, ‘*the morning of the appointment’*.

All quotations presented have been anonymised to ensure individuals or establishments cannot be identified. Participants have been given pseudonyms throughout.

## 3 Results

From the point of secondary care referral, people in prison experience a range of issues not experienced by the wider community. Firstly, we report these issues as themes relating to prisoners accessing secondary care. Secondly, we describe how these relate to the principle of equivalence.

### 3.1 What issues do people in prison experience in trying to access secondary care?

We identified five major themes that described prominent experiences in accessing secondary care, relevant to the principle of equivalence: [1] Security overriding healthcare need or experience [2]; Security creating public humiliation and fear [3]; Difficulties relating to the prison officer's role in medical consultations [4]; Delayed access due to prison regime and transport requirements; and [5] Patient autonomy restricted in management of their own healthcare. Quotation data to support the themes is provided in Table 2, along with comparative summaries of the equivalent experience/process as a community patient. *Italicised* statements represent experiences that are subjectively judged to be inequivalent from the community, whilst those underlined may have some parallels within certain community groups. For example, patients in care homes may be accompanied by care home staff when attending appointments. These staff may be called upon to relay patient clinical information to doctors and may respond on behalf of patients, although this is generally in response to a lack of capacity rather than in the restrictive role associated with prison security.

**Table. 2:** Themes with supporting quotations and comparisons between community and prison experiences of care.

Prisoner experience	Equivalent in community
Security overriding healthcare need or experience	
Patient does not know hospital appointment time or date	*Patient knows appointment time and date*
Patient cannot prepare for appointment (physically or mentally)	*Patient can prepare for appointment*
Patient has no choice over transport means to hospital (can be uncomfortable)	*Patient can exercise choice over appropriate transport to hospital*
Patient has no control over arrival time	*Patient has control over arrival time*
Difficult for patients to access information on their condition	*Patients can use internet/call Consultant secretary freely to access information*

• " […] if I was home I know I'd be in a hospital that day, rather than sitting in my cell, not actually knowing what's wrong with me. […] So, from the point where they told me they booked the appointment, every day I was kind of anxious to know is this the day I'm going to go?" (Dwight) • "I didn't have any time to prepare, […] from the moment that they called me, I've probably got […] 10 min to prepare myself […]I could have been in the middle of cleaning or preparing myself to clean and not found myself in a presentable state that I would want to go " (Dwight) • “…when you go in for an operation […] you need that headspace a day or so before not that morning “Oh you're going for your operation now.” There's no time to prepare” (Leah) • “Then, you get to the hospital […] an hour late […] From my perspective, I feel embarrassed and a pain to them as well, like I'm an inconvenience to them, which it shouldn't be. I'm here, I've done as much as I can. But unfortunately the circumstances we're in and the logistics just don't work. (Adam) • “Because of being in prison and because of not being allowed certain medications there are a few of us that have certain conditions where if we were outside we would be getting different medication to what we get when we're in prison.” (Katy • “You're chained to the officers and you want to discuss your illness or your medication and they don't, like, put a longer chain on and let you have a private conversation with the consultant” (Daryl • “Being in the back of the car with double cuffs, it's uncomfortable, it's frustrating. I think even the most mild mannered person, if you're in a taxi and the car is bumping and grinding and your hands are restrained in that manner, you're going to get a little bit niggly” (Adam)	

Security creating public humiliation and fear	

No privacy for intimate examinations	*Privacy as a gold standard for patient care*
Rushed through public spaces or segregated from general public	*Patients can wait in comfortable waiting areas*
Patient wearing handcuffs	*Patient does not wear handcuffs*

• “I just think that the hospital staff need to look at prisoners as human beings, normal members of the public. […] I don't know if there's a way to allow them to understand that we are also human beings and they're not in any danger as such. […] the prison staff are capable of keeping us under a certain amount of control so they do not need to worry about what's going through their mind, whether he's a murderer or drug dealer or fraudster, whatever it is, they're not there to cause any harm.” (Derek), "…the doctor requested for them to go out with that long chain […] The officer refused. They say no, we have to be here […] it feels uncomfortable. Because I had that long chain I took my tops, everything, my bra and everything out so they left hanging on the chain…" (Flo) • “A lot of women […] are taken to a gynaecologist, and that's supposed to be intimate and private […]. You don't see the gynaecologist on your own. You see them with the two members of staff, handcuffed. […] I have FGM damage to me and they wanted to do reversal and reconstruction surgery. As the doctor was talking to me and […] showing me the computer, these two members of staff were there, and they could see everything. I came back, I was devastated. I was so depressed. I nearly, nearly took my life.“(Anna) • “As soon as you go out of this prison, the whole community, including doctors, public, officers, they are all one and you're the other guy, that everybody looks down on” (Eric), "I think what are they thinking? Am I a rapist? A sex offender? But then I'm none of those things. But I think peoples’ persona when they see a prisoner, oh he must be a murderer or sex offender or rapist" (Bobby), "I felt like a second class citizen. I didn't feel like I was being assessed as a normal member of the public at all. I felt […], well you're not going to die from this injury, let him go. If it's a life or death thing then yes we'll operate, but this guy, nah he's alright. "(Adam • “I felt like a zoo bear that is usually walking with a chain in the nose…” (Eric) • “We eventually found where we were going, […], the hospital staff seemed a little flustered, they didn't know where to seat us. Whether they should seat us in the general public area, or find us a private area to be seated in. They eventually found a back room where we could be seated, which was literally a store cupboard. It had braces and stuff for broken limbs” (Derek) • “As soon as you go up to a desk and speak to a nurse they […] rush you straight through the room, you can't sit in the waiting area with all the people, they just stick you in a room. Then, I've had people come in the room and say, we're going to take you to do this, we're going to put a tube down your throat or whatever. […] I've actually spoken to doctors and nurses and they're quite abrupt with you, it's like I don't matter. I've tried asking them questions and they're not telling me everything. They just want you to hurry up and go basically.” (Aaron)	

Difficulties relating to the prison officer's role in medical consultations	

Prison officers within appointment act as an unchosen companion/support	Patients have the right to choose whether to have someone in an appointment with them and who that is
Prison officers within the consultation compromise patient privacy/confidentiality	Privacy and confidentiality is generally within the remit of patient control (i.e. they can choose whether to have someone else present)
Power in the room – Doctor may address questions and answers to prison officers	Doctor addresses questions and answers to patient
Prison officer dominating conversation/overstepping boundaries of information sharing	*No prison officer in consultation*
Patients are subject to other people's timings and being rushed to complete appointment (by both prison and hospital)	*Patient not rushed by others from their community. Patient may be rushed by hospital due to their timetabling issues but not because of ‘prisoner status’*

• “I've been sitting there and I've been talking about all my reproductive organs and the Officer has gone [said] “So, will she be able to have kids in the future?” “[…] what has that got to do with you?” (Katy) • “When you're a prisoner […], you have no medical confidence. It's open information for everyone that's there and you just get cut out. After a while they don't talk to you, they don't see you, they see authority [of the prison officers] and they bow down to it" (Mohammed) • “Then, once you're there, you've got officers in there listening to what's wrong with you. It's kind of personal. It's a bit embarrassing, you've got two grown arse men there with you. You're trying to speak to a surgeon, there might be things you want to ask that you don't want them to know.." (Bobby) • “Any question that they wanted to ask me was going to the officers. […] So, what type of drugs does he take? Does he do anything else? Does he use the gym? You think hold on a *sec*, I'm right here. But all conversations took place between staff and doctors" (Mohammed) • “The surgeon doesn't know how much power they have at that moment, you know, and I think that they should use their power instead of being intimidated by [prison officers]” (Ian) • “Going out for operations they spoke to the officer “Oh is she allowed sedation?” Hello, I'm the one having the operation it should be me you're asking do I want the sedation, it's got nothing to do with them. Just because they might wanna have a lesser time in the hospital “No, no, no don't give her sedation she'll be alright.”” (Katy)	

Delayed access due to prison regime and transport requirements	

Patients have no control over prison-initiated cancellations, appointment delays and prioritisation of appointments	Appointments not affected by prison regime but may incur delays and cancellations for other reasons
Prison transfers cause interruptions in the care pathway for patients	May incur interruptions for other reasons e.g. voluntary relocation
Patients to be escorted to hospital are prioritised against peers for limited available transfers to hospital	*No equivalent – all patients in community are able to attend their scheduled appointment regardless of the transport arrangements of other patients*

• “I know I missed three appointments due to what we call, spice buses*. If someone goes over and has a medical emergency everything gets cancelled because of the ambulance.” (Adam) • “You've gotta go on a waiting list […] well how far down the list I am. You're at the bottom ‘cause you're not an emergency but to me losing my sight because of that thing was [an emergency]” (Abigail) • “So, if you miss an appointment three times you go back to the bottom of the list. That needs to change for inmates because it's not their fault, they can't, we're not in control of getting to and from the hospital. […] there's a lot of things that come into play on that. If somebody has been fitting overnight and they're out at a hospital that person's appointment is going to get cancelled.” (Katy) • “So I'm sitting there now and waiting and waiting and waiting […] so why haven't I been called for this appointment? Because, I mean, last time it took me five months for me to get from here to the hospital and the condition's getting worse.” (Susan) • “I've had to move establishments while being seen at the hospital. […] which has then led to my whole situation restarting from zero, again which has been very difficult because you're going round in circles and there's no progress on where you should be.” (Ian), *Spice buses refer to emergency transport of a patient to hospital due to use of the psychoactive drug Spice.	

Patient autonomy restricted in management of their own healthcare	

Patient acceptance of a lower standard of care	Patient less likely to settle for perceived lower standard hospital care
Patients cannot manage own condition independently/self care	*Community emphasis on independence and self-care*
Patients cannot book own appointments	*Patients can book own appointments*
Patient anxiety about judgement by staff/public	*Patient may have concerns over judgement by staff on condition but not based on prisoner status*

• “There are some girls that have been in prison since they were young and then if you're going to the hospital with doctors and they're only dealing with the officers, […] you're never having to deal with anything yourself. So, how are you going to cope with that when you get out?" (Lucy), • “it's that independence when you've been on the outside, compared to when you're on the inside it's a bit different, because […] you don't know when you're going. So all that anticipation, you know, when you might get called. When you're least concerned then your name could just be announced that you should show up by the office and then they just take you off there and then…” (Ryan) • "If they said, you're going next week you can think the things you want to ask. Sometimes you forget, when you're put on the spot, you don't know what to ask. But if you were given notice, then you could think to yourself, I need to ask him, this, this and this. Note it all down, so when you go there you can say look, I want […] to ask about this, this and this" (Bobby) • “…it wasn't until last year that I realised that diagnosis means I have this condition for life because when I went out to the hospital the specialist didn't give me all the information that I was supposed to get about the condition I have. He didn't give me all the leaflets that they hand out to every other patient because he was under the inference because I was in prison the prison would sort that side of things out. The prison assumed, oh, because I went out to see the specialist I'd get all the information. […] It's progressive, degenerate and thing and I was given none of that information in the hospital and it was like “Wow, how can you leave a patient so blind?” (Katy) • “If you're not in prison anxiety is […] just a minor thing, it's like you broke your finger or you broke your hand, you go you have a cast on, anxiety is over because you know when that cast comes off you'll be alright. But in prison, it's like, I don't know what's going on, they're not allowed to tell me and the only time I will know is when I actually go see the doctor, […] so you don't know how to feel […]. So the anxiety builds and builds after one visit, and two, and three” (Tony) • "In prison, you find yourself desensitised to certain things. […] You don't make a situation out of certain things. You won't highlight certain things because you're used to a certain standard of care […] So, where I was put in a broom cupboard was wrong to me and my instincts. But because I understand where I am in prison and certain things don't happen as I'd like them, I just decided I'd keep quiet" (Paul) • “[…] and if it went bad, the first thing that you don't want to do is ever go back, I don't want to see that consultant ever again, so I'll miss my appointment next time, but rather than tell anybody […] when they come to […] say that you've got […] a hospital appointment, you end up saying no, I don't want to go. And when that happens, everybody's lost out” (Gavin)	

### 3.2 Security overriding healthcare need or experience

Due to prison security conditions, access to secondary healthcare is operationalised within associated constraints. Prisoners in higher category prisons are not allowed to know the time or date of their hospital appointment in case they make plans to abscond or collect illicit substances. Even in ‘open’ Category D prisons, only trusted prisoners will be allowed to know their appointment time and date. After referral to secondary healthcare, from their perspective they wait for an undetermined period with no indication of when their appointment will take place.

Security conditions present a major challenge to equivalence of care. Participants described being told on the day of their appointment they are going to hospital, and that as appointments are ’impossible to anticipate’ they do not have the opportunity to mentally or physically prepare. Security procedures such as strip searches mean prisoners run late for hospital appointments, resulting in feelings of patient guilt even though this factor is out of their control. Once back in prison, patients may find that medication prescribed or given by the hospital is confiscated, or unavailable for prescription based on prison and healthcare drug policies.

### 3.3 Security creating public humiliation and fear

At hospital, prisoners are handcuffed to officers to ensure they cannot abscond, but this is unrelated to risk of violence. Wearing handcuffs and being accompanied by uniformed officers was described as highly stigmatising, identifying them as a prisoner to other patients and staff, producing negative emotions and fears around hospital attendance. Most people talked about public reactions they receive at the hospital (see Table 2). Participants expressed the wish to convey to hospital staff that they were not violent.

We identified significant inequivalence in physical conditions compared to community patients; during the appointment patients remain handcuffed with prison officers sitting in consultations. This results in distress and compromised privacy for the patient, and can cause logistical issues with testing procedures. People were concerned that because prison officers were not bound by the same duty of confidentiality as medical staff, they may reveal personal information learned in the consultation when back at the prison.

Participants frequently used comparisons to animals, describing their experiences with phrases like *“with that long chain like a dog” (Fay)*, suggesting they undergo feelings of dehumanisation as part of the healthcare process. They rarely feel at the centre of the healthcare journey, with security considerations, logistics and time pressures taking precedence over patient experience.

Some participants felt that clinical staff judged prisoners both in terms of their lifestyle choices (e.g. use of recreational drugs) and the crimes for which they are imprisoned. Several participants questioned whether the care they received was equivalent to community patients but accepted that being in prison meant that differences in care were likely if not inevitable.

### 3.4 Difficulties relating to the prison officer's role in medical consultations

Community patients are generally free to attend hospital appointments unaccompanied, and are typically able to make an informed choice whether to allow someone to accompany them. In closed prisons, patients have no choice but to be accompanied by prison officers, creating a difficult three-way dynamic between the patient, security staff and clinicians. Participants described being accompanied by officers not known to them and of different genders which could be sensitive in certain cases.

Participants stated that hospital staff were frequently compliant with prison officer requests and were reluctant to challenge their perceived authority, despite the clinician having authority within the hospital environment. Many participants noted that during appointments clinicians would often direct their clinical questions and attention at prison officers instead of the patient. Participants described feeling frustrated, upset and patronised, feeling little more than an observer in relation to their own healthcare. Some participants suggested hospital staff seemed unaware that they could challenge prison officers to leave the room through use of a long handcuff chain, despite the obvious benefits this privacy could add.

Participants described that prison officers try to exercise authority over their clinical care. Prison officers operate in shifts, and have an operational requirement to return patients to the prison as quickly as appropriate. This influences them to prefer procedures that reduce overall time spent at hospital, irrespective of whether this constitutes the best care for the patient. For example, if offered a hospital procedure with or without sedation patients report that prison officers may actively try and encourage proceeding without the need for sedation.

While there were many negative secondary care experiences for prisoners, positive experiences were recounted with similar levels of passion but also with sense of surprise and gratitude towards the staff who displayed the behaviour.

*“Every 20 to 30 min they would come and ask me if I'm okay, do I need anything, how am I feeling? I was astonished to get that sort of attention from healthcare. I'm not used to that demand of helpfulness, it was just out of this world. I felt like I had ten mothers in the room […] caring for me, like a mother would for her child. […]. So, that experience I'll never forget, I owe them a lot […]” (Ian)*

Participants were equally grateful to prison officers who made efforts to minimise negative experiences of secondary healthcare appointments. Some prison officers made active efforts to keep the presence of handcuffs discreet in public places. Others actively involved prisoners in their conversations at the hospital, which participants felt helped normalise their presence to the public.

### 3.5 Delayed access due to prison regime and transport requirements

Access to care is an important aspect of equivalence [7, 12] Within prisons, transfers off site to hospital are restricted due to the limited number of prison officers available to act as escorts. When emergencies arise, routine appointments scheduled that day can be cancelled. In effect, prisoners are prioritised against their peers for hospital attendance, inequivalent to community patients who can present at the hospital as required.

Some participants specifically mentioned the concept of lists and their placement on them according to the urgency of their healthcare requirements. When appointments are cancelled and rebooked, patients may effectively be placed back at the bottom of the hospital list as per the UK National Health Service guidelines and made to restart the referral pathway. Prisoners are frequently moved between prisons despite the disruptions in care this may introduce when they have to register and be re-referred afresh with a new local hospital, and start their care pathway from the beginning. The presence of the NHS across England and Wales means that patients can effectively receive the ‘same care’ anywhere in the country, and therefore there is no emphasis on the prison to place the patient on clinical hold and delay their transfer and subsequent appointment cancellations, except in the most critical cases e.g. cancer treatment [13] Due to cancellations, prison-to-prison transfers, and other causes of delay, participants reported delays well over the NHS target of 18 weeks from referral to treatment [14], potentially exacerbating their illness. For those participants aware of this NHS rule it contributed to a sense of unfairness, given that they have no control over cancellations under these circumstances.

### 3.6 Patient autonomy restricted in management of their own healthcare

The opportunity to control healthcare, make significant decisions, and relinquish dependence on others was seen as severely constrained. Participants are unable to book their own appointments, choose the hospital where they will receive treatment or transport themselves. With little time to prepare for an appointment, some participants felt they could not consider the questions they wanted answered by clinicians. This meant they could not easily take control of their own recovery, self-care and overall health on return to the prison. Participants reported that hospital doctors failed to appreciate that prison healthcare departments would not provide in-depth information on their return, nor be able to find their own material from sources typically accessed in the community such as the internet.

### 3.7 Continuity of care

Few participants had experience of trying to ensure continuity of care with hospitals on release from prison, and therefore this topic could not be fully explored within this research. The limited comments that were made referred to: worries about returning to a home far from their current prison and therefore building relationships with a new hospital; default cancellations of hospital appointments upon leaving prisons; and issues with ensuring handover of medical notes from the prison. Participants felt that having their health issues sorted out in prison meant, *“it's one less thing to worry about isn't it?”(Nadia)* on return to the community. Prisons releasing individuals who have an outstanding hospital appointment with the locality to which they are being released, will make efforts to hand over the appointment details to the patient on the day of release from prison. However this cannot happen effectively when there is sudden release from Court.

## 4 Discussion

Despite policies aspiring to equivalence of healthcare for prisoners and the general population, there is a dearth of research on access to care for prisoners. This paper is the first to consider how prisoners experience hospital healthcare, and our analysis relates these experiences directly to healthcare policy with regard to equivalence of care. In common with studies of other prison healthcare environments [15], we found equivalence of care to be highly challenging in hospitals, including physical and practical considerations such as delays in access, but also differences in patient experience such as shame and lack of autonomy.

While security issues are often unavoidable, our data suggests that some prison officers may be reluctant to make proportionate accommodations to help lower risk prisoners conduct medical appointments with dignity, comfort and safety. Despite the availability of guidance for medical professionals on the sensitive and appropriate use of restraints within healthcare settings [16], prisoners rarely reported circumstances where use of restraints was discussed or negotiated by healthcare staff. This represents a complex and challenging area for healthcare. Delayed access to hospital appointments is a harmful consequence of the resource implications associated with transport and security, which could lead to significant adverse health outcomes and legal challenges [17].

The need for autonomy raised in our analysis is circumscribed in international health policy [2–4], where prisoners as patients should be able to make their own informed decisions about healthcare. Autonomy, alongside the principles of justice, beneficence and non-maleficence are the cornerstones of medical ethics [18, 19] Our findings are in consort with other reviews, suggesting that the prison environment is not conducive to promoting autonomy, fundamentally undermining the principle of equivalence [20,21]. The three-way dynamic between security staff, patient and healthcare highlights an area requiring significant improvement and training. The presence of prison officers in hospital appointments undermines both medical confidentiality and autonomy of medical professionals delivering care. The Committee of Ministers of the Council of Europe produced recommendations for delivery of healthcare in prisons which abide by ethical principles. This states in paragraph 13 of the Recommendation No. R(98)7 Concerning the Ethical and Organisational Aspects of Health Care in Prison, *“Medical confidentiality should be guaranteed and respected with the same rigour as in the population as a whole” .*Paragraph 20 further states, *“Clinical decisions and any other assessments regarding the health of detained persons should be governed only by medical criteria. Health care personnel should operate with complete independence within the bounds of their qualifications and competence.”* Although written specifically for healthcare services within prisons, these principles should be applied to healthcare prisoners receive external to the prison environment. The patient-centred approach to shared decision-making is a cornerstone of modern medical practice [22], and it is likely that these adverse experiences are due to lack of knowledge and fears about prison rules rather than intent [16] However, our analyses suggest that the power differential between prisoner and security staff can be exacerbated by hospital staff. Where hospital and prison staff display compassion and understanding this can go some way to mitigating poor experiences at hospitals.

On return to prison prescriptions and/or medication given by the hospital will be subject to a clinical review to determine suitability of the medications in line with ‘Safer Prescribing’ practices in prisons and availability of stock. An understanding amongst community health professionals of the prison prescribing guidance, such as the prison pain formulary [23], may reduce incidents where patients return to the prison with unsuitable prescriptions or medications.

Continuity of care on release could not be fully explored within this research due to the lack of first-hand experience amongst participants, however it remains high on the policy agenda of the health and justice system [24]. Movement of patients between prisons can mean that patients need to re-start their care pathway with a new local hospital, potentially both lengthening the healthcare process and disrupting continuity. Exercising judicious use of ‘clinical hold’ whereby movement of a prisoner between establishments is temporarily restricted, may help address these issues.

Although the setting for this research is English prisons the findings remain relevant to hospitals and prisons in other countries where patients are transported from prisons to community based hospitals for healthcare. The principle of equivalence is enshrined in international policy. Issues that compromise equivalence such as public stigmatisation, reduced privacy and confidentiality, and lack of autonomy are not restricted to English prison systems and should be considered in the design of all prison patient pathways. This paper serves to prompt all healthcare systems to consider whether the care offered to prisons in their locality is comparable to that experienced by a community based patient.

This study has several key strengths. We have been able to gather in-depth qualitative data from a traditionally hard-to-reach, and hard-to-research population. Data collection by peer researchers ensured that participants felt empowered and able to provide honest accounts of their experiences in prison without judgement. The peer researchers were able to provide clarity on terminology used by prisoners, and ensure methods employed were acceptable to this patient group. Furthermore, the wider research team involved in analysis and interpretation of the findings have between them experience of prison healthcare settings, community based hospital settings, and the overarching prison system. This ensured a nuanced view and understanding of practical recommendations that can be made to prisons and hospitals.

Our study also has some limitations. Prison charity peer researchers are not academics and may lack experience gathering qualitative data. They build rapport with participants by acknowledging their own experiences of incarceration, which may lead participants to agree with particular aspects raised in conversation. However, use of peer researchers with lived experience of prison is likely to have gathered a more ‘truthful’ account from participants than would have been possible if academic researchers alone had attempted data collection.

Participation in research activities is ultimately granted by the prison service, on the basis that the participant must not pose a risk to researchers. This research was not undertaken in any high security (Category A) prisons given the additional security restrictions and permissions that would be required. Therefore, those who may experience the closest scrutiny and highest levels of security at hospitals, were not included in the sample.

Few participants in prisons had experience of leaving a prison and trying to continue with hospital care in the community (continuity of care). To understand further the issues surrounding continuity of care for people leaving prison it may be more appropriate to undertake research with ex-offenders residing in the community who are recruited based on experience of hospital care whilst previously imprisoned.

With an ageing and increasingly vulnerable prison population [25, 26], proportionate security measures for offsite healthcare visits should be established to avoid adverse healthcare experiences and exacerbated stigmatisation of prisoners [16]. As long as appointments take place offsite at local hospitals, these issues are unlikely to change significantly. Alternative models such as prison in-reach from hospitals, telemedicine [27] and integrated prison hospitals systems [28] must be vigorously explored to overcome the long waits and cancellations that result in breaches of health system waiting time standards.

Policy makers and senior managers across prison and healthcare jurisdictions should use their power and influence to challenge standards and embedded practices that contribute to this injustice and subsequent health inequalities. Practical actions that can be taken by prison and healthcare staff and policy makers are given in Table 3.

**Table. 3:** Practical advice for professionals.

Theme	Mitigating actions for hospital staff	Mitigating actions for prison staff	Mitigating actions for health care policy makers/ local senior decision makers	Mitigating actions for prison policy makers/ local senior decision makers
*Security overriding healthcare need or experience*	Design services with in-reach/telemedicine components which will allow patients to know their appointment time/date, as there is no risk of escape planning, Understand that patient will not have known they are attending hospital and will not have physically/mentally prepared – allow additional time for questions and empathise if patient appears unsettled/anxious, Understand patient will not have access to specialist information at the prison on their condition – provide leaflets/printouts at the hospital and references to key texts for request by prison libraries, Understand that certain medications will be restricted in prisons (e.g. pregablin) and prescribe within these regimes, Understand patients may not be in possession of medication in prison and reassure patient around medication interval adherence	Encourage patients to write down key questions for hospital staff on the day they are referred to secondary care by prison healthcare, Consider allowing patients more time on the day to physically prepare for their appointment (e.g. bathing), Ensure that the appropriate use of ROTL (Release on Temporary License) and Compassionate Release has been considered	Allow longer appointment slots for patients from prison to ensure all concerns can be addressed within the specified appointment time, Ensure hospitals are aware of and adhere to guidance surrounding restrictions on prescription of medications for people in prison, and patient possession of medication. Ensure clinical staff communicate healthcare/discharge information (medical in confidence) to the prison healthcare team (preferably via Communication Handover sheet), as opposed to relying on relay of clinical information by prison officers, Engage with senior decision makers from local prisons, to collaborate on risk assessment guidance for reduced use of restraints in secondary care facilities	Consider removing restrictions surrounding knowledge of appointment date in closed prisons, for prisoners classified as low escape risk or for those suffering from conditions likely to produce high levels of anxiety during appointment wait, Consider the role of the prison healthcare team in deciding whether a patient should be placed on clinical hold (restricting movement to other prison establishments) if undergoing a period of hospital treatment
*Security creating public humiliation and fear*	Offer patient the choice of an appropriate private waiting area or the public waiting area, Educate clinicians that all patients will wear handcuffs based on security protocols surrounding absconding, and handcuff usage is not based on violence/volatility of the patient, Provide some induction training to staff about patients from prisons to reduce judgement/stigmatisation of this population group	Risk assess in advance whether prison officers can use a long chain/remove handcuffs to leave the consultation room if an intimate exam will likely be taking place, Consider actions prison officers can take to minimise social stigma in public e.g. including patient in conversation, not walking in opposite directions whilst handcuffed to patient	Consider use of a non public entrance route into the hospital for prison patients, to avoid stigmatising public reactions to a patient in handcuffs, Designate a private and appropriate waiting space for patients from prison, Ensure national curriculum for healthcare staff training includes information around patients from secure environments, Consider timing of appointment to allow patient arrival/wait during less busy periods, for example appointments at the beginning or end of a clinic	Consider policy to allow individual risk assessment of whether patient is an escape risk in advance of appointment, and whether rules surrounding handcuffs can be relaxed for hospital attendance
*Difficulties relating to the prison officer's role in medical consultations*	Request use of the long handcuff chain to allow patient privacy for examination/consultation, Address all questions to the patient and not to the accompanying prison officers, Within reason, do not rush patients to leave appointments, Ensure handover of medical information to prison healthcare teams is conducted in a confidential and appropriate manner (medical in confidence), not via prison officers	Remind/educate prison officers on their role within the consultation and the importance of avoiding speaking on behalf of the patient/oversharing information, Within reason, do not rush patients to leave appointments	Work with prisons to understand whether a hospital ‘secure consultation room’ could be established to allow patients to have their appointment without prison officers or handcuffs	Work with hospitals to understand whether a hospital ‘secure consultation room’ could be established to allow patients to have their appointment without prison officers or handcuffs
*Delayed access due to prison regime and transport requirements*	Understand that prisons have operational restrictions surrounding offsite transfers (e.g. limited escorts) and that cancellations/delays occur frequently and can disrupt care pathways, but that these are not in the patient's control., Ensure clinicians do not disengage with prison care provision based on a perceived unwillingness of prison patients to attend hospital appointments. Improve access/referral to treatment times through use of in-reach or telemedicine services, Provide specific appointment slots at the start of the day/expedite appointments for prisoners on arrival at the hospital, which may allow the prison escort to return with another patient for a later appointment the same day	Consider whether additional prison escorts could be provided each day to hospitals	Consider whether standard cancellation policies should apply to the prison system (e.g. starting at bottom of the that waiting list after several cancellations) as this will have been largely out of the patient and prison's control, Clinics should make allowances for the late arrival of patients from prison, given that lateness is generally out of the patient's control, Support local initiatives surrounding telemedicine and in-reach services to prison, by providing adequate operational and financial resource and support to deliver these changes, Work with the prison system to establish protocols for remote digital consultations acceptable to prison governance requirements	Assist individual prison establishments to provide more escorted transfers to hospital each day, Work with the health system to establish protocols for remote digital consultations acceptable to prison governance requirements
*Patients lack autonomy to manage their own healthcare*	Discuss with patient and prison officer the possibilities/limitations of self-management of condition within the prison environment	Ensure instances of poor patient treatment/experience are reported to appropriate channels within the hospital	Work with local prisons to train peers to deliver peer-led advice on accessing and utilising secondary care services	Develop protocols to improve the ability of patients to self-manage their condition within the prison, Work with national partners (for example, in England the national personalised care team) to understand how gold standards of personalised care can be adjusted and applied to prison settings
*Continuity of care*	Appreciate that ex-prisoners attending the local hospital may have commenced a previous care pathway whilst in a prison establishment, and may be confused as to why they need to start this process afresh, If relevant to current clinical care of the patient, enquire with the prison establishment as to how previous secondary care notes could be shared with the hospital to guide current care		Work with prison healthcare teams to establish a process to ensure hospital appointments are not cancelled, and that details are communicated to patients, if the patient is returning to the same local community upon release. Explore the further use of telemedicine appointments to patient's personal devices once they have left the prison, allowing them to honour appointments booked prior to leaving prison e.g. surgical follow ups	Ensure the establishment has robust processes in place for transfer of medical information when a patient moves prison/ returns to the community

In complement to the research findings presented in this article the authors have developed a five-minute animation narrated by current prisoners to be used as engagement tool for discussions as to how services can be made more inclusive for prisoners, freely available from the UCL Institute of Epidemiology and Health Care website.

Our study has underlined that access to hospital healthcare is not enough to ensure that policy standards are upheld, and this is dependant on both prison and healthcare organisations adapting to the inequalities imposed by necessary security conditions. This is an example of the Inverse Care Law, whereby those most in need of healthcare are least able to access it [29]. People in prison often come from the most deprived areas of the community, and are subject to significant health inequalities. Achieving equivalence of care for prisoners requires investment of a higher level of resource than for community patients, to create prison-focussed solutions and pathways that exceed, rather than fail, community standards of care [30]. These investments will transfer out into the community, improving the health of society overall [31]. Prison healthcare remains an evolving landscape, and hospital care is one of many areas requiring further research with respect to equivalence.

## Declaration of Competing Interest

CE reports grants from Wellcome Trust, grants from Surrey Heartlands Health and Care Partnership, grants from NIHR during the conduct of the study. All other authors declare no conflict of interest.

## Appendix Supplementary materials

Image, application 1

Image, application 2

## References

[1] Aldridge R.W., Story A., Hwang S.W., Nordentoft M., Luchenski S.A., Hartwell G. (2018). Morbidity and mortality in homeless individuals, prisoners, sex workers, and individuals with substance use disorders in high-income countries: a systematic review and meta-analysis. Lancet.

[2] Swiss Association of Medical Sciences SAoM. Ethics Support in Medicine - Recomendations 2012 [Available from:http://www.samw.ch/de/Ethik/Ethische-Unterstuetzung-in-der-Klinik.html.

[3] United Nations. Principles of Medical Ethics Relevant to the Role of Health Personnel, Particularly Physicians, in the Protection of Prisoners and Detainees Against Torture and Other Cruel, Inhuman, or Degrading Treatment or Punishment. 1982.

[4] United Nations (1988). Body of principles for the protection of all persons under any form of detention or imprisonment.

[5] Leaman J., Richards A.A., Emslie L., O'Moore E.J (2017). Improving health in prisons–from evidence to policy to implementation–experiences from the UK. Int J Prison Health.

[6] Till A., Forrester A., Exworthy T (2014). The development of equivalence as a mechanism to improve prison healthcare. J R Soc Med.

[7] RCGP (2018). Equivalence of care in secure environments in the UK, London.

[8] HM Prison Service and National Health Service The Future Organisation Of Prison Health Care (1999). Home office and national health service executive working group, London.

[9] Ismail N., de Viggiani N (2018). How do policymakers interpret and implement the principle of equivalence with regard to prison health? A qualitative study among key policymakers in England. J Med Ethics.

[10] Guest G., Namey E., McKenna K (2016). How many focus groups are enough? Building an evidence base for nonprobability sample sizes. Field Methods.

[11] Ritchie J.S,.L (1994). Qualitative data analysis for applied policy research.

[12] Assembly UG (2015). Nelson Mandela rules.

[13] Hartley B.Benias Razumas v Ministry of Justice [2018]EWHC 215 (QB) UK2018 [

[14] NHS (2013). The handbook to the NHS constitution.

[15] Bretschneider W., Elger B.S. (2014). Expert perspectives on Western European prison health services: do ageing prisoners receive equivalent care?. J Bioeth Inquiry.

[16] Association BM (2009). The medical role in restraint and control: custodial settings.

[17] Benius Razumas v Ministry of Justice [press release]. 2018.

[18] Gillon R (1994). Medical ethics: four principles plus attention to scope. BMJ.

[19] Beauchamp T., Childress J.F. (1989). Principles of biomedical ethics.

[20] Jotterand F., Wangmo T. (2014). The principle of equivalence reconsidered: Assessing the relevance of the principle of equivalence in prison medicine. Am J Bioeth.

[21] Niveau G. (2007). Relevance and limits of the principle of "equivalence of care" in prison medicine. J Med Ethics.

[22] Stewart M.Patient-centered medicine: transforming the clinical method: Radcliffe Publishing; 2003.

[23] England N. (2017). Pain management formulary for prisons: the formulary for acute.

[24] England N., Justice Ha (2018). editor. Guidance for improving continuity of care between prison and the community.

[25] Mann N (2016). Doing harder time? The experiences of an ageing male prison population.

[26] Ministry of Justice. Safety in Custody Statistics Bulletin, England and Wales: Deaths in Prison Custody to March 2019, Assaults and Self-harm to December 2018. 2019.

[27] Edge C., Black G., King E., George J., Patel S., Hayward A. (2019). Improving care quality with prison telemedicine: the effects of context and multiplicity on successful implementation and use. J Telemed Telecare.

[28] Raimer B.G., Stobo J.D. (2004). Health care delivery in the Texas prison system: the role of academic medicine. JAMA.

[29] Tudor Hart J. (1971). The inverse care law. Lancet.

[30] Lines R. (2006). From equivalence of standards to equivalence of objectives: The entitlement of prisoners to health care standards higher than those outside prisons. Int J Prison Health.

[31] Ismail N. (2019). Rolling back the prison estate: the pervasive impact of macroeconomic austerity on prisoner health in England. J Public Health.

[32] Estelle v. Gamble Kristi M. McKinnon In: Encyclopedia of Prisons & Correctional Facilities Edited by: Mary Bosworth DOI: 10.4135/9781412952514.n115. https://sk.sagepub.com/reference/prisons/n115.xml.

