# Supplementary material for: Secondary care clinicians and staff have a key role in delivering equivalence of care for prisoners: A qualitative study of prisoners’ experiences
Source: eClinicalMedicine. 2020 Jun 21;24:100416. doi: 10.1016/j.eclinm.2020.100416 (PMC7525130; doi:10.1016/j.eclinm.2020.100416)
Supplement: Supplementary file 2 [file mmc2.docx]

# Research in context

Evidence before this study

There is widespread agreement that there are barriers to making prison healthcare equivalent to that in the community, given the closed nature of the prison environment and the operational focus on delivering the orders of the court. Prisoners frequently experience suboptimal healthcare experiences, such as long delays to access hospital care. Recent research has called for reform in the way healthcare is delivered in prisons to ensure it does not form part of the punishment associated with deprivation of liberty.

There is a lack of research on the experiences within secondary care services external to the prison specifically. A search of prominent literature databases (Pubmed, Embase, date range 2000-2019) did not reveal any research currently published on this topic.

Added value of this study

To our knowledge this is the first study to provide new information on the experiences of prisoners who receive secondary care off-site at local hospitals. Further to this it seeks to frame these experiences in line with the principle of equivalence, as compared to the expected experience of a patient in the general community. This adds value to existing prison research focused on healthcare services delivered within the prison and outcomes of patients within prisons. This also adds value with respect to the limited research on prisoner experiences, or healthcare delivered to prisoners in hospital settings external to the prison. The value of this study is in the interpretation of the experiences of prisoners at secondary care to produce actionable points staff can take to improve their services for prisoners.

**Implications of all the available evidence**

Despite accessing the same secondary healthcare services as community patients, prisoners’ experiences of secondary care in England are inequivalent to those a community patient would likely receive. The current system for provision of secondary healthcare to prisoners in England does not allow for comparable quality or access to healthcare, due to security and operational constraints, and a lack of awareness amongst healthcare staff. Action from frontline staff and policymakers, from both hospital and prison systems, is required to provide more humanising and accessible secondary care for this underserved population group.
